# AKAP1 contributes to impaired mtDNA replication and mitochondrial dysfunction in podocytes of diabetic kidney disease

**DOI:** 10.7150/ijbs.73493

**Published:** 2022-06-13

**Authors:** Jun Feng, Zhaowei Chen, Yiqiong Ma, Xueyan Yang, Zijing Zhu, Zongwei Zhang, Jijia Hu, Wei Liang, Guohua Ding

**Affiliations:** 1 Division of Nephrology, Renmin Hospital of Wuhan University, Wuhan, Hubei, China.; 2 Nephrology and Urology Research Institute of Wuhan University, Wuhan, Hubei, China.

**Keywords:** AKAP1, Larp1, PKC, mtDNA replication, mitochondria, podocyte, diabetic kidney disease

## Abstract

Podocyte injury is involved in the onset and progression of diabetic kidney disease (DKD) and is associated with mitochondrial abnormalities. Defective mitochondrial DNA (mtDNA) replication results in mitochondrial dysfunction. However, whether podocyte mtDNA replication is impaired in DKD is still unclear. A-kinase anchoring protein 1 (AKAP1) is localized in the outer mitochondrial membrane (OMM) and acts as a regulator and conductor of mitochondrial signals. Herein, we investigated the role of AKAP1 in high glucose-induced mtDNA replication. Decreased mtDNA replication and mitochondrial dysfunction occurred in podocytes of DKD. AKAP1 expression was up-regulated, and protein kinase C (PKC) signaling was activated under hyperglycemic conditions. AKAP1 recruited PKC and mediated La-related protein 1 (Larp1) phosphorylation, which reduced the expression of mitochondrial transcription factor A (TFAM), a key factor in mtDNA replication. In addition, mtDNA replication, mitochondrial function and podocyte injury were rescued by knocking down AKAP1 expression and the PKC inhibitor enzastaurin. In contrast, AKAP1 overexpression worsened the impairment of mtDNA replication and podocyte injury. In conclusion, our study revealed that AKAP1 phosphorylates Larp1 via PKC signaling activation to decrease mtDNA replication, which accelerates mitochondrial dysfunction and podocyte injury in DKD.

## Introduction

Diabetic kidney disease (DKD) is the most common microvascular complication in patients with diabetes and is often accompanied by mass proteinuria 1. Current treatments for DKD are limited, and DKD pathogenesis and treatment are undergoing further investigation 2. Podocytes participate in the formation of the glomerular filtration barrier, and podocyte injury correlates with proteinuria, which plays a key role in DKD progression 3. It is relatively clear that podocyte injury contributes to DKD. The kidney is an energy-demanding organ, and mitochondria are energy-generating organelles that maintain cellular homeostasis. The main characteristics of mitochondrial dysfunction include abnormal mitochondrial dynamics, decreases in adenosine triphosphate (ATP) generation and mitochondrial membrane potential (MMP), reactive oxygen species (ROS) overproduction, and mitochondrial autophagy disorders. Previous studies have shown that excessive mitochondrial fission and ROS production occur in podocytes of DKD 4, 5. However, the regulatory mechanisms of mitochondrial abnormalities remain unknown.

Mitochondrial DNA (mtDNA) is independent of nuclear DNA and encodes subunits involved in the mitochondrial respiratory chain, which mediates to proceed oxidative phosphorylation (OXPHOS), including ND1-6, ND4L, COX I-III, cyt-b, ATPase6 and ATPase8 6. mtDNA replication is closely related to mitochondrial metabolism 7, 8. Mitochondrial transcription factor A (TFAM), which is located in mitochondria, is necessary for mtDNA replication and mitochondrial biogenesis 9. However, mtDNA lacks a mature self-repair system and is susceptible to mutations triggered by external disturbances 10. mtDNA damage leads to cellular dysfunction and disease occurrence 11, 12. It has been shown that hyperglycemia can induce mtDNA strand lesions and reduce mitochondrial complex activities 13. An increase in mtDNA copy number in plasma is associated with chronic inflammation in type 2 diabetic patients 14. Although mitochondrial dysfunction has been verified in podocytes, whether mtDNA replication is abnormal in DKD remains unclear. The correlation between mtDNA replication and mitochondrial function needs to be examined.

A‐kinase anchoring protein 1 (AKAP1) is an anchoring protein localized on the outer mitochondrial membrane (OMM), that recruits various molecules to regulate mitochondrial functions, including mitochondrial metabolism and dynamics 15, 16. AKAP1 plays an important role in mitochondrial biogenesis and function. It has been reported that MDI, Drosophila AKAP homolog, is essential for mtDNA replication and female fertility in the ovary 17. Increased AKAP1 expression induced by hyperglycemia exacerbates mitochondrial damage in podocytes of DKD 4. AKAP1 mediates second messenger signaling to facilitate local mitochondrial protein synthesis, and impacts the translation and activity of the electron transport chain (ETC) complex 18. However, the specific regulatory mechanism of AKAP1 in mtDNA replication remains incompletely understood.

In this study, we investigated the role of AKAP1 in mtDNA replication in podocytes, and further elucidated the underlying molecular mechanisms to provide theoretical basis for podocyte injury in DKD.

## Methods and materials

### Animal models

Eight-week-old male Sprague-Dawley rats were purchased from SJA Laboratory Animals (Hunan, China). The rats were randomly divided into the control group (n=6) and the diabetic group (n=6). The diabetic group was treated with streptozotocin (STZ, 65 mg/kg of body weight) by intraperitoneal injection, and 0.1 mol/L citrate buffer (pH=0.45) was administered to the control group 4. Blood glucose was examined every two weeks. Rats with blood glucose levels exceeding 16.7 mM were considered diabetic 19. At the age of 20 weeks, the rats were sacrificed, and the kidneys were collected for further study.

Seven-week-old male db/db mice and matched db/m mice were purchased from CAVENS Laboratory Animals (Jiangshu, China). Mice aged eight weeks underwent intrarenal lentivirus administration 20. The lentiviral vector pLVX-shRNA2-ZSGreen-T2A-Puro packaging shAKAP1 (Lv-shAKAP1) or the negative control (Lv-CTL) were administered to the kidneys. The mice were randomly separated into four groups: in group 1, db/m mice were injected with Lv-CTL (n=5); in group 2, db/m mice were injected with Lv-shAKAP1 (n=5); in group 3, db/db mice were injected with Lv-CTL (n=5); and in group 4, db/db mice were injected with Lv-shAKAP1 (n=5). Twenty-four-hour proteinuria, the urinary albumin creatinine ratio (UACR), blood glucose, body weight and other indicators were tested every two weeks after injection. At the age of 16 weeks, the kidneys and blood samples were harvested for histological and biochemical analyses.

All animal experimental procedures were approved by the Ethics Committee for the Experimental Use of Animals of Renmin Hospital of Wuhan University.

### Pathological studies

The kidneys were fixed in 4% paraformaldehyde at room temperature, and wrapped in a wax block. The kidney sections were stained with hematoxylin-eosin (HE) and periodic acid-Schiff (PAS) to assess pathological changes. Renal tissues were fixed in 2.5% glutaraldehyde, and ultrastructural changes were assessed by electron microscopy.

### Cell culture

Conditionally immortalized human podocytes were provided by Dr. Moin A. Saleem (Academic Renal Unit, Southmead Hospital, Bristol, UK). The podocytes were cultured in a 33 °C incubator and then differentiated in a 37 °C incubator for one week. Specific cell culture was performed as previously described 5. After the indicated stimulation, the cells were processed for subsequent experiments.

### siRNA transfection

Transfection of siRNA was conducted by using HiPerFect transfection reagent according to the manufacturer's instructions. A total of 2×10^5^ cells were seeded in each well of a six-well plate. Then, the cells were transfected with a mixture containing 2 μl of siRNA solution (10 μM) and 10 μl of transfection reagent for two days under normal conditions. The AKAP1 target sequence was TGGGCTTGGCACTCAAGTCAA.

### Adenovirus transfection

Podocytes were transfected with 0.5 μl of human AKAP1 overexpression adenovirus (2×10^12^ vp/ml) for 6 h, and then washed with PBS buffer and cultured in a 37 °C incubator.

### EdU staining

EdU incorporation was used to determine DNA replication in cells 21. 2 ml of EdU working solution (10 μM) was added to each well of a six-well plate and incubated for 3 h in a 37 °C incubator. Next, 4% paraformaldehyde was used to fix the cells for 15 min. Then, washing solution and 0.3% Triton permeation solution were used to wash the samples, which were incubated with click additive solution in the dark for 30 min. After being washed with washing solution, the slides were mounted.

### Western blotting

Protein sample preparation was performed as previously described 5. Equal amounts of protein were separated by SDS-PAGE and then transferred to PVDF membranes (Sigma, USA). The membranes were blocked with 5% nonfat milk for 1 h and incubated with primary antibodies (AKAP1, 1:500; Larp1, 1:500; PKC, 1:500; BAX, 1:1000; GAPDH, 1:1000; Phosphorylation antibody, 1:1000) overnight at 4 °C. Next, the membranes were incubated with HRP-conjugated goat anti-rabbit/mouse IgG (H+L) secondary antibodies, for 1 h. The proteins were examined with ECL reagent and a Bio-Rad imaging system.

### Immunoprecipitation

Cells were cultured in a petri dish (NEST, China) with a diameter of 10 cm. After the appropriate treatments, proteins were extracted and incubated with 5 μl of primary antibodies and 30 μl of protein A+G agarose suspension beads with rotation overnight at 4 °C. The beads were washed 3 times and boiled in loading buffer at 100 °C for 10 min. Next, the proteins were separated by SDS-PAGE. Additional steps the same as those for Western blotting.

### Immunohistochemistry

Paraffin-embedded kidney sections were dewaxed, antigen retrieval was performed, and the sections were blocked and dyed as described previously 22. The samples were incubated with primary antibodies (AKAP1, 1:100; Larp1, 1:100; PKC, 1:100; TFAM, 1:100).

### Immunofluorescence assay

An immunofluorescence assay was performed as previously described 22. The samples were incubated with primary antibodies (AKAP1, 1:100; Larp1, 1:100; PKC, 1:100; TFAM, 1:100; TOM20, 1:200; Synaptopodin, 1:100).

### Quantitative real-time polymerase chain reaction (RT-PCR)

Total RNA was extracted by Trizol reagent, and a Nanodrop was used to measure the RNA concentration. Next, cDNA was synthesized by a reverse transcription kit. The mRNA expression levels of the target genes were determined by a real-time fluorescence-based quantitative PCR instrument (Bio-Rad, USA). The primer sequences were designed according to PrimerBank** (Table 1)**. The operation steps were performed in accordance with the protocol.

### Measurement of mitochondrial ROS, MMP and ATP production

The assays to analyze mitochondrial ROS production, MMP and ATP synthesis in cells were performed as previously described 4.

### DHE staining

Fresh frozen sections of the kidneys were prepared and incubated with 20 mM DHE dye solution under normal temperature and dark conditions for 1 h. After being washed with PBS buffer, the slides were mounted, and the fluorescent images were collected rapidly.

### Apoptosis assay

The degree of cell apoptosis was assessed by flow cytometry (BD FACS Calibur, USA) according to the instructions of Annexin V-ADD Apoptosis Detection Kit I. After resuspending the cells in 100 μl of 1× binding buffer, 5 μl of Annexin V-ADD and 5 μl of PE were added to each sample and incubated for 15 min at room temperature in the dark. The cells were resuspended in 400 μl 1× binding buffer before the apoptosis assay was performed.

### Statistical analysis

All data represent at least 3 independent experiments, and statistical analyses were performed by GraphPad Prism 9. The statistical significance of differences was determined by Student's two-tailed t test (*p* < 0.05 indicated statistical significance).

## Results

### Impaired mtDNA replication and mitochondrial dysfunction in podocytes under high glucose (HG) conditions

**Figure 1:**
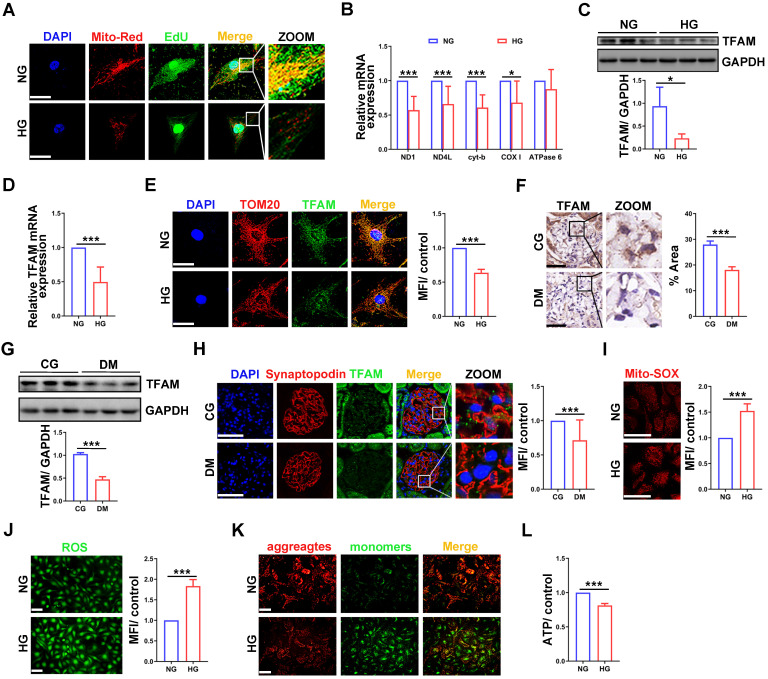
** Impaired mtDNA replication and mitochondrial dysfunction in podocytes under HG conditions. (A)** EdU and MitoTracker red staining in normal and HG-treated podocytes. **(B)** Quantitative PCR analysis of mtDNA, including ND1, ND4L, cyt-b, COX I and ATPase6, in the two groups. (Scale bars, 50 µm. n=6, *p < 0.05, ***p < 0.001). **(C)** Western blot and quantitative analysis of TFAM in normal and HG-treated podocytes. (n=3, *p < 0.05). **(D)** Quantitative PCR analysis of TFAM in the two groups. (n=6, ***p < 0.001). **(E)** Immunofluorescence (IF) and semiquantitative analysis of TFAM in the two groups. (Scale bars, 50 µm. n=6, ***p < 0.001). (F) Immunohistochemistry (IHC) and semiquantitative analysis of TFAM expression in the glomeruli of STZ-induced diabetic rat group and control group. (Scale bars, 50 µm. n=6, ***p < 0.001). **(G)** Western blot and quantitative analysis of TFAM expression in the renal cortex of two groups. (n=3, ***p < 0.001). **(H)** IF and quantitative analysis of TFAM expression in the glomeruli of the two groups. (Scale bars, 50 µm. n=6, ***p < 0.001). **(I)** Analysis of mitochondrial ROS production in normal and HG-treated podocytes. (Scale bars, 100 µm, n=6, ***p < 0.001). **(J)** Analysis of ROS generation in the two groups. (Scale bars, 100 µm. n=6, ***p < 0.001). (K) MMP assay via JC-1 staining in the two groups. (Scale bars, 100 µm.) **(L)** Semiquantitative analysis of ATP production in the two groups. (n=6, ***p < 0.001).

To investigate whether mtDNA replication was impaired in HG-treated podocytes, we performed EdU and MitoTracker red staining. Compared with that in the normal group, the fluorescence intensity was decreased in HG-treated podocytes **(Figure 1A)**. The mRNA expression of mtDNA-related genes, including ND1, ND4L, cyt-b and COX Ⅰ, was also decreased in HG-treated podocytes **(Figure 1B)**. TFAM is the key factor in mtDNA replication 23. TFAM expression was significantly decreased in HG-treated podocytes **(Figure 1C-E)**. Similarly, TFAM expression was decreased in the glomeruli and podocytes of STZ induced diabetic rats **(Figure 1F-H)**. These results suggested that mtDNA replication was decreased in podocytes of DKD. Mitochondrial function was evaluated to confirm whether it correlated with mtDNA replication. ROS production was increased, and MMP and ATP synthesis were decreased in HG-treated podocytes **(Figure 1I-L)**. These results showed that HG treatment impaired mtDNA replication and mitochondrial dysfunction in podocytes.

### Increased AKAP1 expression in diabetic glomeruli and HG-treated podocytes

**Figure 2:**
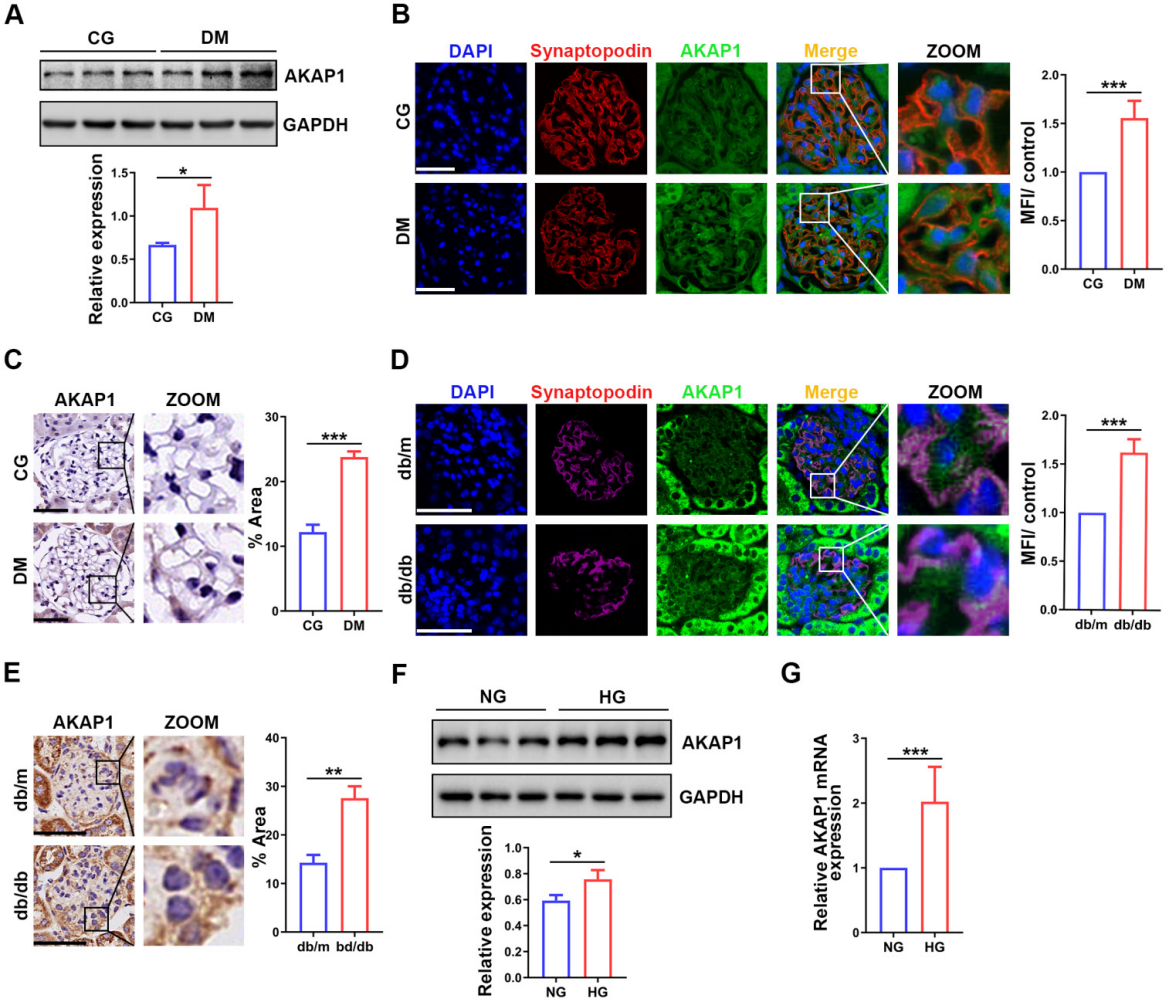
** Changes of AKAP1 expression in podocytes of DKD. (A)** Western blot and quantitative analysis of AKAP1 in the renal cortex of STZ-induced diabetic rat group and control group. (n=3, *p < 0.05). **(B)** IF and quantitative analysis of AKAP1 in glomeruli of the two groups. (Scale bars, 50 µm. n=6, ***p < 0.001). **(C)** IHC and quantitative analysis of AKAP1 in the glomeruli of the two groups. (Scale bars, 50 µm. n=6, ***p < 0.001). **(D)** IF and quantitative analysis of AKAP1 in the glomeruli of the diabetic mouse group and control group. (Scale bars, 50 µm. n=6, ***p < 0.001). **(E)** IHC and quantitative analysis of AKAP1 in the glomeruli of the two groups. (Scale bars, 50 µm. n=6, ***p < 0.001). **(F)** Western blot and quantitative analysis of AKAP1 in the normal and HG-treated cell groups. (n=3, *p < 0.05). **(G)** Quantitative PCR analysis of AKAP1 in the two cell groups. (n=6, ***p < 0.001).

To evaluate the role of AKAP1 in podocytes of DKD, we analyzed the expression of AKAP1. AKAP1 expression was upregulated in the glomeruli and podocytes of diabetic rats and mice **(Figure 2A-E)**, similar to HG-treated podocytes **(Figure 2F-G)**. Collectively, these results verified the increased expression of AKAP1 in podocytes of DKD. This result was consistent with our previous findings 4.

### Knocking down AKAP1 improved renal function and mtDNA replication in diabetic mice

**Figure 3:**
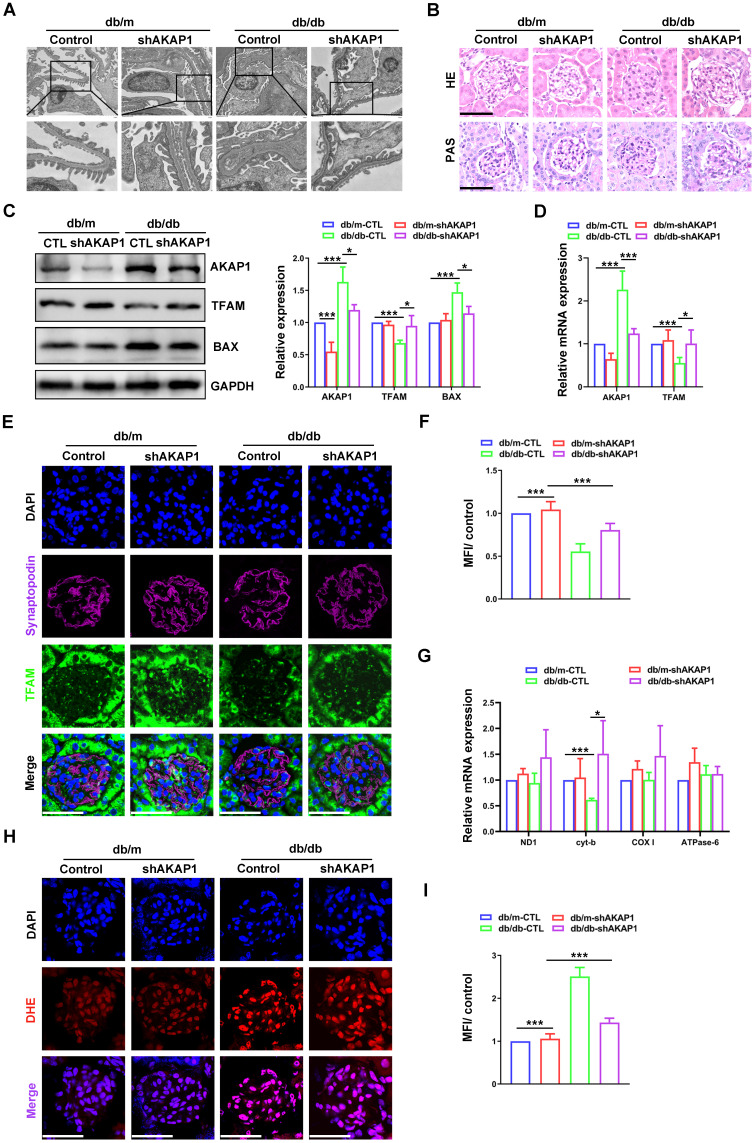
** Effects of knocking down AKAP1 on renal function and mtDNA replication in the kidneys of diabetic mice. (A)** Representative transmission electron microscopy images of podocyte foot processes and the glomerular basement membrane in control and diabetic mice. **(B)** Representative HE and PAS staining of glomeruli in the different groups. (Scale bars, 50 µm). **(C)** Representative Western blot and quantitative analysis of AKAP1, TFAM and BAX in the four groups. (n=3, *p < 0.05, ***p < 0.001). **(D)** Quantitative PCR analysis of AKAP1 and TFAM in the four groups. (n=3, *p < 0.05, ***p < 0.001). **(E-F)** Double fluorescent labeling of TFAM and synaptopodin and quantitative analysis in the glomeruli of the different groups. (Scale bars, 50 µm. n=6, ***p < 0.001). **(G)** Quantitative PCR analysis of mtDNA including ND1, cyt-b, COX I and ATPase6 in four groups. (n=3, *p < 0.05, ***p < 0.001). **(H-I)** DHE staining and quantitative analysis of the glomeruli in the four groups. (Scale bars, 50 µm. n=6, ***p < 0.001).

To further investigate the relationship between the increase in AKAP1 expression and impaired mtDNA replication, the expression of AKAP1 was knocked down in the kidneys of diabetic mice. Renal function and podocytes were markedly impaired in diabetic mice. Twenty-four-hour urinary protein, UACR and serum creatinine were alleviated in diabetic mice with AKAP1 knockdown **(Figure SA-C)**. However, urine volume, blood glucose and body weight were not obviously improved **(Figure SD-F)**. Moreover, damage to the glomerular structure was ameliorated, including foot process fusion and mesangial hyperplasia **(Figure 3A-B)**. Increased TFAM expression and decreased AKAP1 and BAX expression were examined in the glomeruli of AKAP1-knockdown diabetic mice **(Figure 3C-D)**. We used fluorescent staining to analyze localization and identified increased TFAM expression in the podocytes of diabetic mice with AKAP1 knockdown **(Figure 3E-F)**. The gene expression of cyt-b, a mtDNA gene involved in mitochondrial complex Ⅲ, was significantly increased by AKAP1 knockdown **(Figure 3G)**. AKAP1 deficiency reduced ROS production in the glomeruli of diabetic mice **(Figure 3H-I)**. These results showed that AKAP1 knockdown alleviated mtDNA replication impairment.

### AKAP1 deficiency ameliorated mtDNA replication impairment and mitochondrial dysfunction in HG-treated podocytes

**Figure 4:**
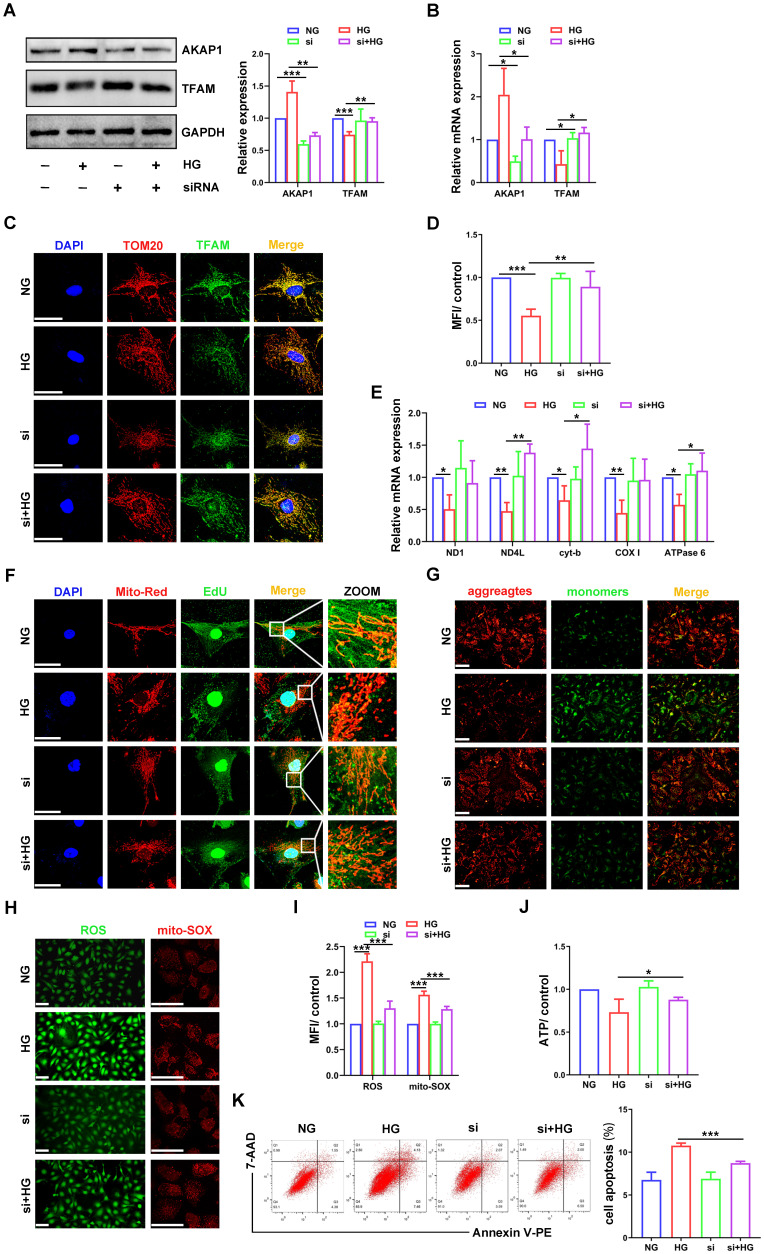
** Effects of AKAP1 deficiency on mtDNA replication and mitochondrial function in HG-treated podocytes. (A)** Representative Western blot and quantitative analysis of AKAP1 and TFAM in the NG, HG, AKAP1 siRNA and HG+AKAP1 siRNA groups. (n=3, *p < 0.05, **p < 0.01, ***p < 0.001). **(B)** Quantitative PCR analysis of AKAP1 and TFAM in the four groups. (n=3, *p < 0.05). **(C-D)** Double fluorescent labeling and quantitative analysis of TFAM in the four groups. (Scale bars, 50 µm. n=6, **p < 0.01, ***p < 0.001). **(E)** Quantitative PCR analysis of mtDNA, including ND1, ND4L, cyt-b, COX I and ATPase6, in the four groups. (n=3, *p < 0.05, **p < 0.01). **(F)** EdU and MitoTracker red staining in podocytes of the four groups. (Scale bars, 50 µm). **(G)** MMP assay via JC-1 staining in the different groups. (Scale bars, 100 µm). **(H-I)** Semiquantitative analysis of mitochondrial ROS production in the different groups. (Scale bars, 100 µm. n=6, ***p < 0.001). **(J)** Semiquantitative analysis of ATP production in the different groups. (n=4, *p < 0.05). **(K)** Quantitative analysis of cell apoptosis by flow cytometry in the different groups. (n=3, ***p < 0.001).

Next, we examined the relationship between AKAP1 expression and changes in mtDNA replication in cultured podocytes. AKAP1 expression was silenced successfully by siRNA transfection **(Figure 4A-B)**. Compared to that in HG-treated podocytes, TFAM expression was upregulated by AKAP1 silencing **(Figure 4A-D)**. AKAP1 silencing enhanced mtDNA replication, and the gene expression of ND4L, cyt-b, and ATPase6 was increased significantly **(Figure 4E-F)**. Next, we evaluated the effect of improving mtDNA replication on mitochondrial function. In the presence of AKAP1 silencing, ROS production was decreased, and MMP and ATP synthesis were significantly increased **(Figure 4G-J)**. Cell apoptosis was also decreased in AKAP1 siRNA-treated podocytes **(Figure 4K)**. These results indicated that AKAP1 deficiency ameliorated the impairment in mtDNA replication and mitochondrial function in HG-treated podocytes.

### AKAP1 overexpression exacerbated alterations in mtDNA replication and mitochondrial dysfunction in HG-treated podocytes

**Figure 5:**
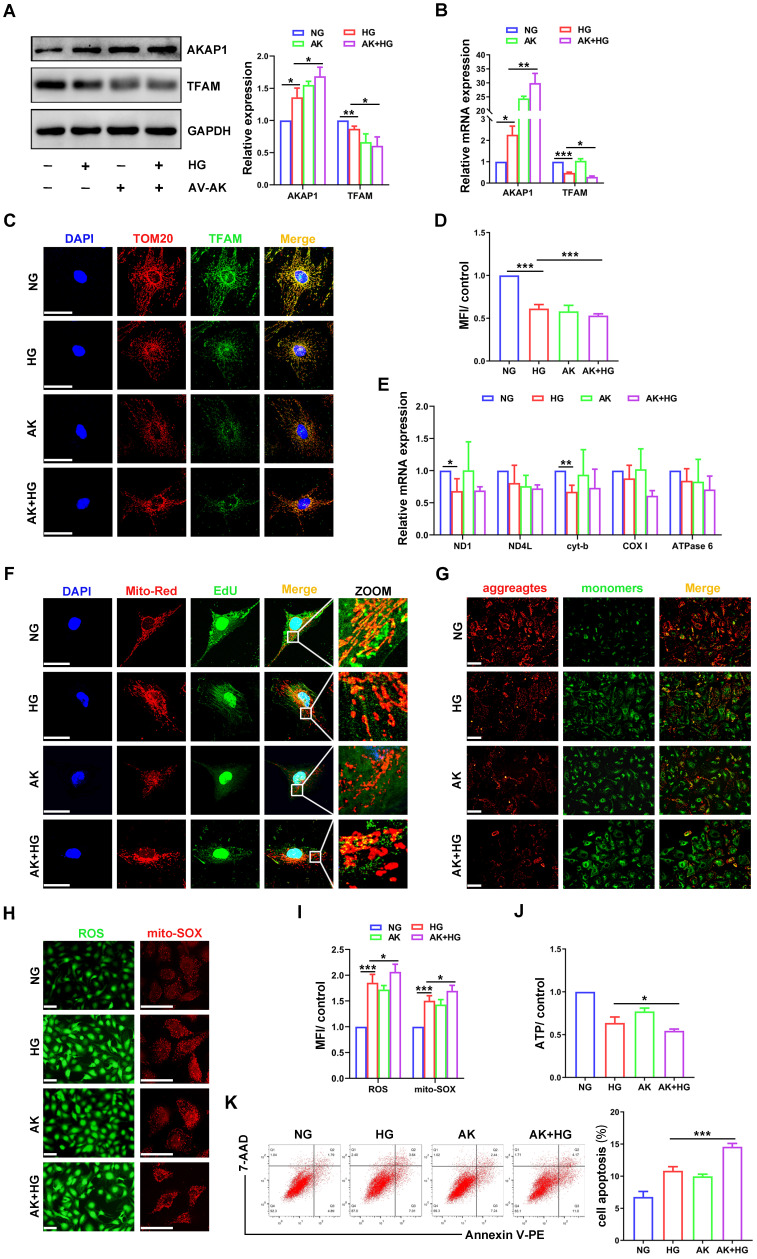
** Effects of AKAP1 overexpression on mtDNA replication and mitochondrial function in HG-treated podocytes. (A)** Representative Western blot and quantitative analysis of AKAP1 and TFAM in the NG, HG, AV-AKAP1 and HG+AV-AKAP1 groups. (n=3, *p < 0.05, **p < 0.01). **(B)** Quantitative PCR analysis of AKAP1 and TFAM in the four groups. (n=3, *p < 0.05, **p < 0.01, ***p < 0.001). **(C-D)** Double fluorescent labeling and quantitative analysis of TFAM in the four groups. (Scale bars, 50 µm. n=6, ***p < 0.001). **(E)** Quantitative PCR analysis of mtDNA including ND1, ND4L, cyt-b, COX I and ATPase6 in the four groups. (n=3, *p < 0.05, **p < 0.01). **(F)** EdU and MitoTracker red staining in podocytes of the four groups. (Scale bars, 50 µm). **(G)** MMP assay via JC-1 staining in the different groups. (Scale bars, 100 µm). **(H-I)** Semiquantitative analysis of mitochondrial ROS production in the different groups. (Scale bars, 100 µm. n=6, *p < 0.05, ***p < 0.001). **(J)** Semiquantitative analysis of ATP production in the different groups. (n=4, *p < 0.05). **(K)** Quantitative analysis of cell apoptosis by flow cytometry in the different groups. (n=3, ***p < 0.001).

To examine whether AKAP1 overexpression could affect mtDNA replication in podocytes, we increased AKAP1 expression by exogenous transfection **(Figure 5A-B)**. Compared to that in the HG group, AKAP1 overexpression further reduced TFAM expression **(Figure 5A-D)**, and mtDNA replication was decreased **(Figure 5E-F)**. Mitochondrial function was impaired, such as ROS overproduction and decreased MMP and ATP synthesis **(Figure 5G-J)**. Podocyte apoptosis was also increased by AKAP1 overexpression **(Figure 5K)**. These results indicated that AKAP1 overexpression exacerbated the impairment in mtDNA replication and mitochondrial dysfunction in HG-treated podocytes.

### Larp1 phosphorylation was increased in podocytes of DKD

**Figure 6:**
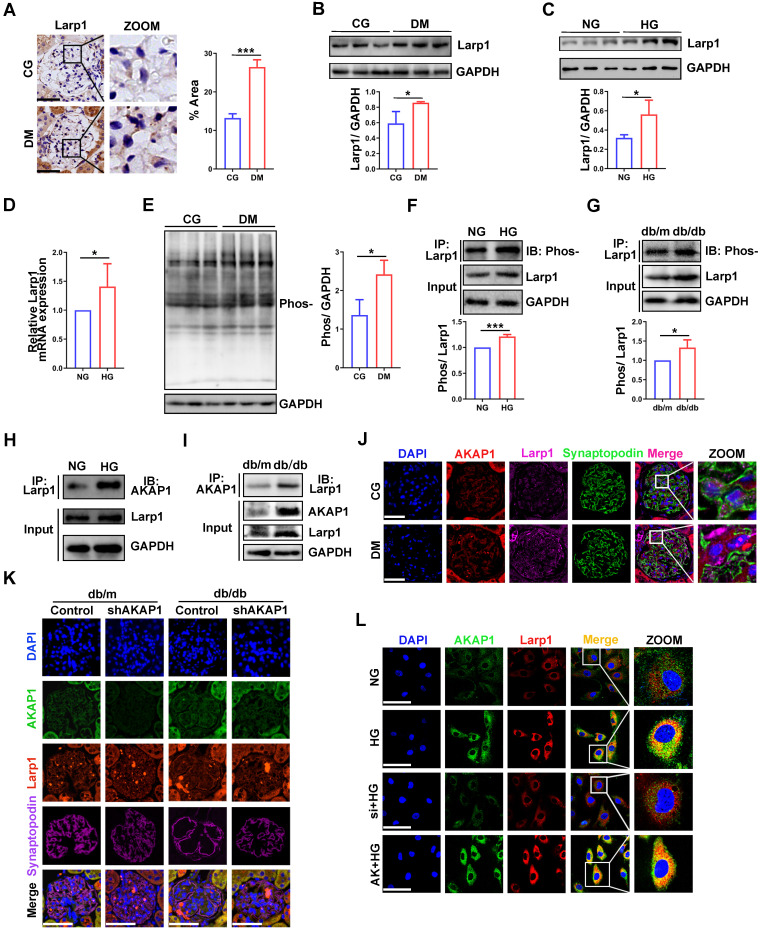
** Relationship between AKAP1 and Larp1 in podocytes of DKD. (A)** IHC and quantitative analysis of Larp1 in the glomeruli of the STZ-induced diabetic rat group and control group. (Scale bars, 50 µm. n=6, ***p < 0.001). **(B)** Representative Western blot and quantitative analysis of Larp1 in renal cortex of the two groups. (n=3, *p < 0.05). **(C)** Western blot and quantitative analysis of Larp1 in the normal and HG-treated cell groups. (n=3, *p < 0.05). **(D)** Quantitative PCR analysis of Larp1 in the two cell groups. (n=6, ***p < 0.001). **(E)** Western blot and quantitative analysis of total protein phosphorylation in the control and diabetic rat groups. (n=3, *p < 0.05). **(F)** Immunoprecipitation (IP) and quantitative analysis of Larp1 phosphorylation in normal and HG-treated podocytes. (n=3, ***p < 0.001). **(G)** IP and quantitative analysis for Larp1 phosphorylation in the renal cortex of control and diabetic mice. (n=3, *p < 0.05). **(H)** Representative Western blot of AKAP1 and Larp1 by IP in normal and HG-treated podocytes. **(I)** Representative Western blot of AKAP1 and Larp1 by IP in db/m and db/db mice. **(J)** Triple fluorescent labeling of AKAP1, Larp1 and synaptopodin in the glomeruli of the control and diabetic rat groups. (Scale bars, 50 µm). **(K)** Triple fluorescent labeling of AKAP1, Larp1 and synaptopodin in the glomeruli of the control and diabetic mouse groups. (Scale bars, 50 µm). **(L)** Double fluorescent labeling of AKAP1 and Larp1 in podocytes of the different groups. (Scale bars, 100 µm).

Larp1 specifically recognizes and stabilizes multiple mRNAs to post-transcriptionally regulate gene expression 24. Larp1 expression was upregulated in the glomeruli of diabetic rats **(Figure 6A-B)**, similar to HG-treated podocytes **(Figure 6C-D)**. There is a correlation between the phosphorylation of Larp1 and its molecular activity 25. Therefore, we further measured the level of phosphorylated Larp1. Overall protein phosphorylation level was increased in the kidney cortex of diabetic rats **(Figure 6E)**. Phosphorylated Larp1 was also increased in HG-treated podocytes and the kidney cortex of diabetic mice **(Figure 6F-G)**. These data confirmed the upregulation of phosphorylated Larp1 in podocytes of DKD.

### Increased interaction of AKAP1 and Larp1 in podocytes of DKD

To evaluate the relationship between AKAP1 and Larp1 in podocytes of DKD, we verified that AKAP1 bound with Larp1 in HG-treated podocytes and the kidney cortex of diabetic animals **(Figure 6H-J)**. AKAP1 knockdown reduced the interaction of AKAP1 with Larp1 **(Figure 6K-L)**. In contrast, AKAP1 overexpression increased their binding **(Figure 6L)**.

### AKAP1 recruits PKC to phosphorylate Larp1

**Figure 7:**
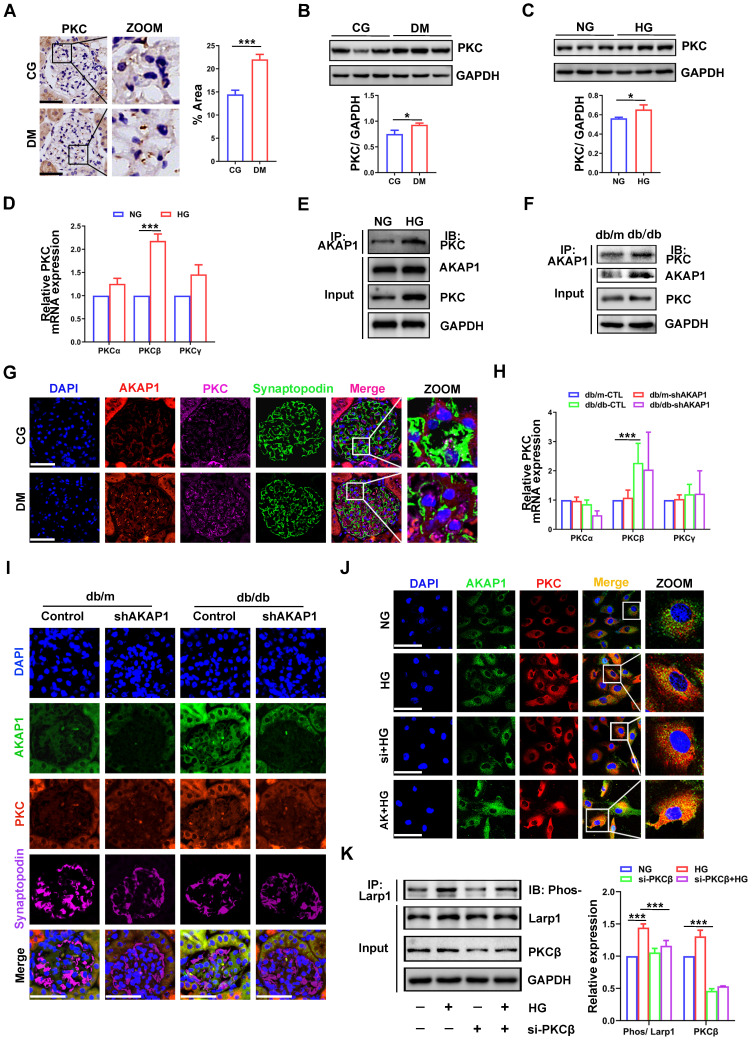
** Role of PKC in mediating Larp1 phosphorylation in podocytes of DKD. (A)** IHC and quantitative analysis of PKC in the glomeruli of the STZ-induced diabetic rat group and control group. (Scale bars, 50 µm. n=6, ***p < 0.001). **(B)** Representative Western blot and quantitative analysis of PKC in the two animal groups. (n=3, *p < 0.05). **(C)** Western blot and quantitative analysis of PKC in the normal and HG-treated cell groups. (n=3, *p < 0.05). **(D)** Quantitative PCR analysis of PKCα, PKCβ and PKCγ in the two animal groups. (n=6, ***p < 0.001). **(E)** Representative Western blot of AKAP1 and PKC by IP in the two groups. **(F)** Representative Western blot of AKAP1 and PKC by IP in db/m and db/db mice. **(G)** Triple fluorescent labeling of AKAP1, PKC and synaptopodin in the glomeruli of control and diabetic rat groups. (Scale bars, 50 µm). **(H)** Quantitative PCR analysis of PKCα, PKCβ and PKCγ in the control and diabetic mouse groups. (n=3, ***p < 0.001). **(I)** Triple fluorescent labeling of AKAP1, PKC and synaptopodin in the glomeruli of different groups. (Scale bars, 50 µm). **(J)** Double fluorescent labeling of AKAP1 and PKC in podocytes of the different groups. (Scale bars, 50 µm). **(K)** IP and quantitative analysis of Larp1 phosphorylation in normal and PKCβ siRNA-treated podocytes. (n=3, ***p < 0.001).

Then, we further examined the relationship between AKAP1 and phosphorylated Larp1. Surprisingly, PKC played a key role in the interaction between AKAP1 and phosphorylated Larp1. PKC signaling is activated and mediates the kidney injury in DKD 26, 27. In our study, we verified that PKC expression was increased in glomeruli and podocytes of DKD **(Figure 7A-C)**. In particular, the gene expression of PKCβ, a subtype of PKC, was significantly increased in HG-treated podocytes** (Figure 7D, H)**, indicating that PKCβ might initiate Larp1 phosphorylation in podocytes of DKD. Under HG stimulation, AKAP1 recruited more PKC **(Figure 7E-G)**. AKAP1 knockdown reduced its interaction with PKC **(Figure 7I-J)**, but AKAP1 overexpression increased their binding **(Figure 7J)**. To investigate whether PKCβ was the key factor mediating Larp1 phosphorylation, we silenced PKCβ by siRNA transfection, and the level of phosphorylated Larp1 was significantly decreased in HG-treated podocytes **(Figure 7K)**. These results suggested that AKAP1 might recruit PKC to phosphorylate Larp1 in podocytes of DKD.

### Enzastaurin attenuated the impairment of mtDNA replication and mitochondrial dysfunction in HG-treated podocytes

**Figure 8:**
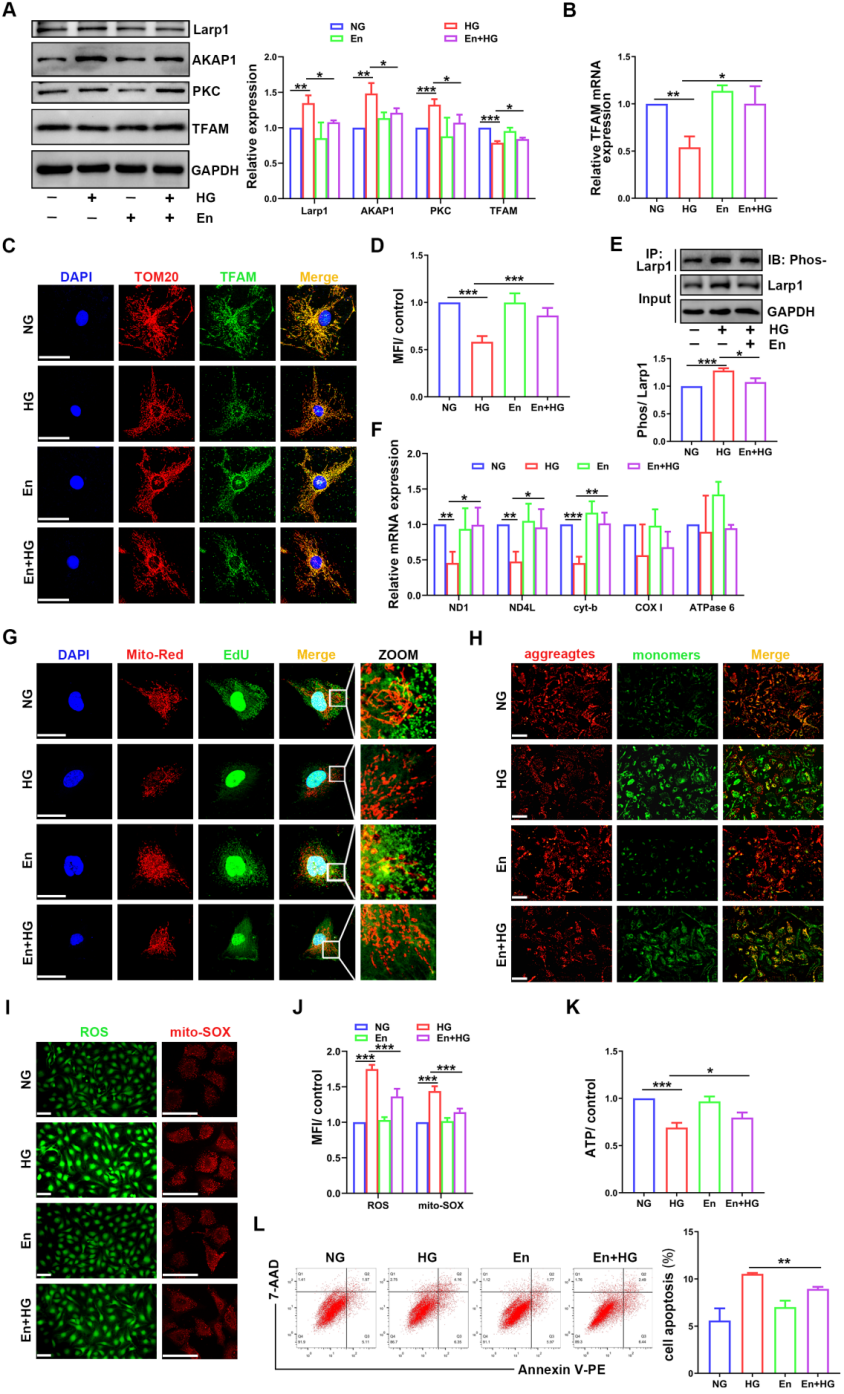
** Effects of enzastaurin on HG-treated podocytes. (A)** Representative Western blot and quantitative analysis of Larp1, AKAP1, PKC and TFAM in NG, HG, enzastaurin and HG+enzastaurin-treated cell groups. (n=3, *p < 0.05, **p < 0.01, ***p < 0.001). **(B)** Quantitative PCR analysis of TFAM in podocytes of the four groups. (n=3, *p < 0.05, **p < 0.01). **(C)** Double fluorescent labeling of TFAM and TOM20 in the four groups. (Scale bars, 50 µm). **(D)** Quantitative analysis of TFAM fluorescence intensity in podocytes of the four groups. (n=6, ***p < 0.001). **(E)** IP and quantitative analysis of Larp1 phosphorylation in normal, HG and enzastaurin-treated podocytes. (n=3, ***p < 0.001). **(F)** Quantitative PCR analysis of mtDNA, including DN1, ND4L, cyt-b, COX I and ATPase6, in the four groups. (n=3, *p < 0.05, **p < 0.01, ***p < 0.001). **(G)** EdU and MitoTracker red staining in podocytes of the four groups. (Scale bars, 50 µm). **(H)** MMP assay via JC-1 staining in the different groups. **(I-J)** Semiquantitative analysis of mitochondrial ROS production in the different groups. (Scale bars, 100 µm. n=6, ***p < 0.001). **(K)** Semiquantitative analysis of ATP production in the different groups. (n=4, *p < 0.05, ***p < 0.001). **(L)** Quantitative analysis of cell apoptosis by flow cytometry in the different groups. (n=3, **p < 0.01).

To confirm the role of PKC in mtDNA replication, the inhibitor enzastaurin was used in HG-treated podocytes. Enzastaurin inhibited PKCβ activity, and PKC inhibition enhanced TFAM expression **(Figure 8A-D)**. The phosphorylation of Larp1 was decreased by enzastaurin treatment **(Figure 8E)**. Moreover, mtDNA replication and the gene expression levels of ND1, ND4L and cyt-b were increased in enzastaurin-treated podocytes **(Figure 8F-G)**. Furthermore, we evaluated mitochondrial function. Compared to that in the HG-treated group, ROS production was decreased, and MMP and ATP synthesis were improved in the enzastaurin-treated group **(Figure 8H-K)**, which was inconsistent with cell apoptosis **(Figure 8L)**. These results showed that PKC inhibition by enzastaurin played a protective role by improving mtDNA replication and mitochondrial function in HG-treated podocytes.

## Discussion

This study demonstrated that mtDNA replication was impaired in podocytes of DKD, which resulted in mitochondrial dysfunction and cell apoptosis. Under hyperglycemic conditions, increased AKAP1 expression and activated PKC signaling occurred in the kidney. AKAP1 promoted Larp1 phosphorylation via PKC signaling activation, which affected TFAM expression, further leading to impaired mtDNA replication and mitochondrial dysfunction, including increased ROS production and reduced ATP synthesis. Taken together, these results suggested that AKAP1/Larp1 signaling mediated the impairment in mtDNA replication and mitochondrial dysfunction via PKC activation in podocytes of DKD.

DKD, which is the major cause of chronic kidney disease (CKD) and end stage renal disease (ESRD), is closely related to mitochondrial dysfunction, including mitochondrial dynamics, bioenergetics, biosynthesis, mitophagy, oxidative stress and mtDNA disorders 28-30. Likewise, we confirmed impaired ATP synthesis and ROS overproduction in DKD. mtDNA encodes 13 subunits involved in the ETC, which is associated with ATP synthesis and ROS production 31. Damaged mtDNA is released into the circulation and causes an inflammatory response 32. In DKD patients, urinary mtDNA levels were negatively correlated with the estimated glomerular filtration rate (eGFR) and intrarenal mtDNA and positively correlated with interstitial fibrosis 33. Consistent with urinary mtDNA levels, an increase in mtDNA copy number was observed in the peripheral blood of DKD patients 34. Under diabetic conditions, excessive mtDNA filtration through the kidney may be involved in chronic renal inflammation 35. These studies have revealed that abnormal mtDNA is involved in DKD. Aberrant mtDNA could be considered a biomarker of DKD. It may also be interesting to explore the correlation between mtDNA leakage into peripheral blood and urine and low intracellular mtDNA amounts in DKD.

mtDNA replication contributes to cellular homeostasis, and a reduction in mtDNA copy number is associated with the progression of many diseases, including diabetes, neurodegeneration, tumors, and aging 36-39. A recent study showed that the protein kinase B (AKT)/mammalian target of rapamycin (mTOR) signaling pathway was involved in regulating mtDNA levels and enhancing cellular oxidase activity and mitochondrial OXPHOS^40^. Activation of the adenosine monophosphate-activated protein kinase (AMPK)/silent information regulator 1 (SIRT1)/peroxisome proliferator-activated receptor γ coactivator-1α (PGC-1α) pathway could also promote mtDNA content and mitochondrial biosynthesis 41. TFAM binds to promoter sites on mtDNA, mediates mtDNA transcription and translation, and maintains mtDNA stability 42. Ca^2+^ flux is regulated by TFAM through mitochondria-endoplasmic reticulum interactions and signals to the nucleus, resulting in the alleviation of metabolic disorders 43. TFAM can also reduce mtDNA damage and cytoplasmic mtDNA release, thereby reducing mitochondrial damage and inflammatory responses 44. Previous studies have shown that the expression of the ETC subunits CO1, CO2, CO3, ATP6 and ATP8 was reduced, which was accompanied by decreased TFAM expression in the renal cortex of diabetic rats, and activation of the cAMP response element binding protein (CREB)/PGC-1α/TFAM signaling pathway improved mitochondrial biogenesis and function by increasing ETC subunit expression 45. Consistently, we verified decreased mtDNA replication and TFAM expression in podocytes of DKD in present study. This abnormality may lead to disrupted integrity of ETC, especially mitochondrial complex III, resulting in reduced ATP synthesis and increased ROS production. Decreased mtDNA replication in podocytes is involved in the progression of DKD. It has been reported that there is a correlation between mtDNA replication and mitochondrial fission 46. Mitochondrial fusion and fission can alter the mixed contents required for mtDNA replication, thereby regulating mtDNA replication and distribution 47, 48. Mitofusin1/2 (MFN1/2) also regulates the expression of TFAM, which maintains mtDNA levels 49. Exploring the regulatory mechanism between mtDNA replication and mitochondrial dynamics will be a noteworthy topic.

AKAP1 provides a platform for numerous mitochondrial signaling pathways. We recently demonstrated that AKAP1 could recruit dynamin-related protein 1 (Drp1) to promote mitochondrial fission, causing mitochondrial dysfunction and podocyte injury in DKD 4, 50. AKAP1 deletion attenuated diet-induced obesity and insulin resistance by promoting fatty acid oxidation and thermogenesis in brown adipocytes 51. AKAP1 interacts with the NADH-ubiquinone oxidoreductase subunit to maintain mitochondrial complex Ⅰ activity and ATP production 52. In this study, we found that increased AKAP1 expression was related to impaired mtDNA replication in podocytes of DKD, and AKAP1 deficiency promoted mtDNA replication and mitochondrial function, indicating that AKAP1 is essential for mtDNA replication and mitochondrial function.

Larp1, a novel RNA-binding protein, stabilizes mRNA structure and coordinates mRNA translation and protein synthesis 53. As a nonmitochondrial protein, Larp1 regulates mitochondrial protein translation. Larp1 is correlated with intact OXPHOS capacity, and Larp1 knockdown partially reduces oxygen consumption and decreases MT-CO1 and MT-CO2 expression, which further influences the integrity of the mitochondrial respiratory chain 54. The phosphorylation state regulates mRNA translation and subsequent ribosome biogenesis 55. Increased phosphorylation of Larp1 is responsible for the progression of nonalcoholic steatohepatitis and hepatocellular carcinoma 56. Phosphorylation of Larp1 by PTEN-induced kinase 1 (PINK1) disrupted mitochondrial local protein synthesis and reduced mtDNA replication 57. The MDI-Larp complex is essential for the synthesis of partial nuclear-encoded mitochondrial proteins via cytoplasmic ribosomes on the OMM, and the MDI deficiency inhibits mtDNA replication in the ovary 17. Interestingly, we observed an increase in the interaction of AKAP1 and Larp1 in podocytes under hyperglycemic conditions. Phosphorylated Larp1 was also increased in podocytes of DKD, which led to reduced TFAM expression and mtDNA replication, further triggering mitochondrial dysfunction. These results suggest that the AKAP1/Larp1 pathway plays an important role in regulating mtDNA replication.

PKC signaling activation is associated with mitochondrial damage 58. DKD pathological injury and progression are also accompanied by PKC signaling activation 26. The PKC inhibitor ruboxistaurin can stabilize the eGFR and reduce urinary albumin in DKD patients, indicating the protective effect of PKC inhibition on delaying DKD progression 59. PKC directly phosphorylates its target molecule, further compromising its function 60. Similarly, we demonstrated that Larp1 was phosphorylated by PKC, and PKC inhibition reversed this phosphorylation, which improved TFAM expression, mtDNA replication and mitochondrial function in podocytes of DKD. PKC inhibition might be a new strategy for DKD treatment.

This study was performed using animal models and cultured cells. However, these findings need to be further validated in podocyte AKAP1 conditional knockout mice. The role of Larp1 and the identity of the phosphorylation sites in podocytes of DKD could be further examined.

In conclusion, we have demonstrated that the AKAP1/Larp1 pathway mediates abnormal mtDNA replication and mitochondrial function via PKC signaling activation in podocytes of DKD. These results reveal a new molecular mechanism for the progression of DKD and may provide a theoretical basis for new treatment strategies.

## Highlights

- Decreased mtDNA replication and mitochondrial dysfunction occurs in podocytes of diabetic kidney disease;
- Impaired mtDNA replication aggravates mitochondrial dysfunction and podocyte apoptosis;
- AKAP1 is located in mitochondrial outer membrane and regulates mtDNA replication and mitochondrial function;
- AKAP1 phosphorylates Larp1 to limit TFAM expression by recruiting activated PKC.

## Supplementary Material

Supplementary figure.

## Figures and Tables

**Table 1:** The sequence of qPCR primers

Target gene	Species	Forward (5'→3')	Reverse (5'→3')
AKAP1	Human	CCTTGCCGAAGATCAGAGTCC	TGCTGGAGAATAGTACACCCTTT
Larp1	Human	ACACAAGTGGGTTCCATTACAAA	CTCCGCGATTGGCAGGTAT
PKCα	Human	ATGTCACAGTACGAGATGCAAAA	GCTTTCATTCTTGGGATCAGGAA
PKCβ	Human	AAACCTTGTACCTATGGACCCC	CCCAATCCCAAATCTCTACTGAC
PKCγ	Human	AGCCACAAGTTCACCGCTC	GGACACTCGAAGGTCACAAAT
TFAM	Human	GGGACAGAGGTGGCTCAACA	CAATCACAACTGGAACCCGC
ND1	Human	CCTACGGGCTACTACAACCC	GCGATGGTGAGAGCTAAGGT
ND4L	Human	ATAACCCTCAACACCCACTCC	TGGAGATTGAGACTAGTAGGGC
cyt-b	Human	CCCATCCAACATCTCCGCAT	GAGGCGTCTGGTGAGTAGTG
COX I	Human	ATACCAAACGCCCCTCTTCG	TGTTGAGGTTGCGGTCTGTT
ATPase6	Human	ACCACAAGGCACACCTACAC	TATTGCTAGGGTGGCGCTTC
GAPDH	Human	GGAGTCCACTGGCGTCTTCA	GTCATGAGTCCTTCCACGATACC
AKAP1	Mouse	AAGCTATGACCCCACCACTG	CGCAACAGCTATCCACTGAA
Larp1	Mouse	GCCTACACCTGGAGAGATAGC	GGCAACTTACGAGCAGGCT
PKCα	Mouse	AGAGGTGCCATGAGTTCGTTA	GGCTTCCGTATGTGTGGATTTT
PKCβ	Mouse	CCCTCAATCCAGAGTGGAATGA	CCAAATGACAGAGATCCCATGAA
PKCγ	Mouse	AAGTTCACCGCTCGTTTCTTC	GCTACAGACTTGACATTGCAGG
TFAM	Mouse	CAAAGGATGATTCGGCTCAGG	TCGACGGATGAGATCACTTCG
ND1	Mouse	CCCTACCAATACCACACCCATT	GGGCTACGGCTCGTAAGCT
cyt-b	Mouse	GAGGTTGGTTCGGTTTTGG	GTTTTGAAAGGGTGGGTGAC
COX I	Mouse	CAGACCGCAACCTAAACACA	TTCTGGGTGCCCAAAGAAT
ATPase6	Mouse	CCATAAATCTAAGTATAGCCATTCCAC	AGCTTTTTAGTTTGTGTCGGAAG
GAPDH	Mouse	AACTTTGGCATTGTGGAAGG	ACACATTGGGGGTAGGAACA

**Table 2:** Key resources

Reagents	Source	Identifier
Rabbit monoclonal anti-AKAP1 antibody	Cell Signaling Technology	Cat# 5203
Mouse monoclonal anti-Larp1 antibody	Santa Cruz Biotechnology	Cat# sc-515873
Rabbit polyclonal anti-Larp1 antibody	Proteintech	Cat# 13708-1-AP
Mouse monoclonal anti-PKC antibody	Abcam	Cat# ab31
Mouse monoclonal anti-PKC antibody	Abcam	Cat# ab23511
Rabbit polyclonal anti-TFAM antibody	Proteintech	Cat# 22586-1-AP
Rabbit polyclonal anti-Phospho-(Ser/Thr) Phe antibody	Cell Signaling Technology	Cat# 9631
Mouse monoclonal anti-BAX antibody	Proteintech	Cat# 60267-1-Ig
Mouse monoclonal anti-TOM20 antibody	Santa Cruz Biotechnology	Cat# sc-17764
Mouse monoclonal anti-Synaptopodin antibody	Santa Cruz Biotechnology	Cat# sc-515842
Rabbit polyclonal anti-GAPDH antibody	GeneTex	Cat# GTX100118
HRP-Goat Anti-mouse IgG(H+L)	AntGene	ANT019
HRP-Goat Anti-rabbit IgG(H+L)	AntGene	ANT020
Alexa Fluor 488 Donkey anti Mouse IgG(H+L)	AntGene	ANT023S
Alexa Fluor 594 Donkey anti Rabbit IgG(H+L)	AntGene	ANT030S
Protein A+G agarose suspension beads	Calbiochem	Cat# IP05
Trizol reagent	Takara	T9108
cDNA synthesis kit	Thermo Fisher Scientific	Cat# K1622
SybrGreen qPCR mastermix	DBI Bioscience	DBI-2044
Dihydroethidium dye solution	Yeasen	50102ES02
BeyoClick EdU kit with Alexa Fluor 488	Beyotime	C0071S
ROS assay kit	Beyotime	S0033M
ATP assay kit	Beyotime	S0026
Mitochondrial membrane potential assay kit with JC-1	Beyotime	C2006
MitoSOX red mitochondrial superoxide indicator	YEASEN	40778ES50
Mitotracker Red CMXRos	YEASEN	40741ES50
PE Annexin V apoptosis detection kit Enzastaurin	BD Biosciences Targetmol	559763 T6280-1
AKAP1 adenovirus	Vigene Biosciences	VH899251
AKAP1 siRNA	QIAGEN	Cat# 1027416
PKCβ siRNA	Santa Cruz Biotechnology	Cat# sc-29450
HiPerFect Transfection Reagent	QIAGEN	Cat# 301705
DNA Transfection Reagent	Roche	XTG9-RO

## Abbreviations

AKAP1: A-kinase anchoring protein 1
AKT: protein kinase B
AMPK: Adenosine monophosphate-activated protein kinase
ATP: adenosine triphosphate
CKD: chronic kidney disease
CREB: cAMP response element binding protein
DKD: diabetic kidney disease
Drp1: dynamin-related protein 1
eGFR: estimated glomerular filtration rate
ETC: electron transport chain
HE: hematoxylin-eosin
HG: high glucose
IF: Immunofluorescence
IHC: Immunohistochemistry
IP: Immunoprecipitation
Larp1: La-related protein 1
MFN: mitofusin
MMP: mitochondrial membrane potential
mtDNA: mitochondrial DNA
mTOR: mammalian target of rapamycin
OMM: outer mitochondrial membrane
OXPHOS: oxidative phosphorylation
PAS: periodic acid‐Schiff
PGC-1α: peroxisome proliferator-activated receptor γ coactivator-1α
PINK1: PTEN-induced kinase 1
PKC: protein kinase C
ROS: reactive oxygen species
SIRT1: silent information regulator 1
STZ: streptozotocin
TFAM: mitochondrial transcription factor A
UACR: urinary albumin creatinine ratio
