# Supplementary figures and images for: AKAP1 contributes to impaired mtDNA replication and mitochondrial dysfunction in podocytes of diabetic kidney disease
Source: Int J Biol Sci. 2022 Jun 13;18(10):4026–42. doi: 10.7150/ijbs.73493 (PMC9274505; doi:10.7150/ijbs.73493)

**A**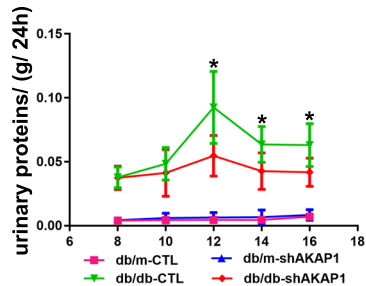**B**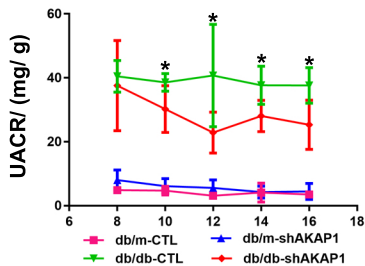**C**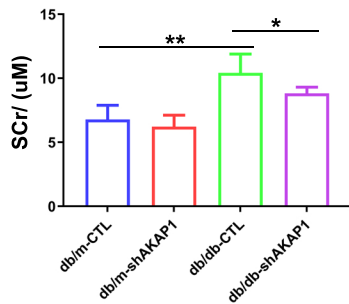**D**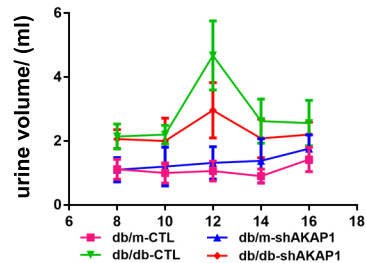**E**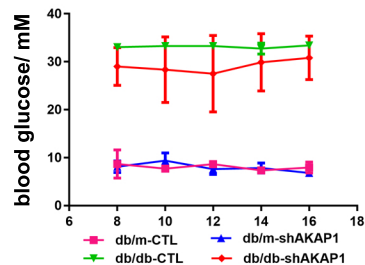**F**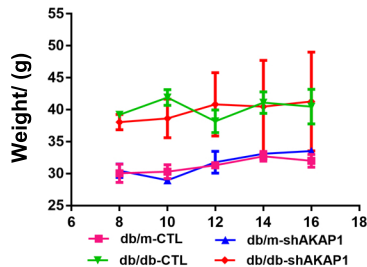

Supplement: Supplementary file 1 — Supplementary figure. [file ijbsv18p4026s1.pdf]
